# Use and Effects of Patient Access to Medical Records in General Practice Through a Personal Health Record in the Netherlands: Protocol for a Mixed-Methods Study

**DOI:** 10.2196/10193

**Published:** 2018-09-21

**Authors:** Maria MT Vreugdenhil, Rudolf B Kool, Kees van Boven, Willem JJ Assendelft, Jan AM Kremer

**Affiliations:** 1 Scientific Center for Quality of Healthcare Radboud Institute for Health Sciences Radboud University Medical Center Nijmegen Netherlands; 2 Department of Primary and Community Care Radboud Institute for Health Sciences Radboud University Medical Center Nijmegen Netherlands

**Keywords:** health records, patient access to records, patient participation, personal, decision making, shared, medication adherence, patient-centered care, self-management

## Abstract

**Background:**

In the Dutch health care system, general practitioners hold a central position. They store information from all health care providers who are involved with their patients in their electronic health records. Web-based access to the summary record in general practice through a personal health record (PHR) may increase patients’ insight into their medical conditions and help them to be involved in their care.

**Objective:**

We describe the protocol that we will use to investigate the utilization of patients’ digital access to the summary of their medical records in general practice through a PHR and its effects on the involvement of patients in their care.

**Methods:**

We will conduct a multilevel mixed-methods study in which the PHR and Web-based access to the summary record will be offered for 6 months to a random sample of 500 polypharmacy patients, 500 parents of children aged <4 years, and 500 adults who do not belong to the former two groups. At the patient level, a controlled before-after study will be conducted using surveys, and concurrently, qualitative data will be collected from focus group discussions, think-aloud observations, and semistructured interviews. At the general practice staff (GP staff) level, focus group discussions will be conducted at baseline and Q-methodology inquiries at the end of the study period. The primary outcomes at the patient level are barriers and facilitators for using the PHR and summary records and changes in taking an active role in decision making and care management and medication adherence. Outcomes at the GP staff level are attitudes before and opinions after the implementation of the intervention. Patient characteristics and changes in outcomes related to patient involvement during the study period will be compared between the users and nonusers of the intervention using chi-square tests and t tests. A thematic content analysis of the qualitative data will be performed, and the results will be used to interpret quantitative findings.

**Results:**

Enrollment was completed in May 2017 and the possibility to view GP records through the PHR was implemented in December 2017. Data analysis is currently underway and the first results are expected to be submitted for publication in autumn 2019.

**Conclusions:**

We expect that the findings of this study will be useful to health care providers and health care organizations that consider introducing the use of PHR and Web-based access to records and to those who have recently started using these.

**Trial Registration:**

Netherlands Trial Registry NTR6395; http://www.trialregister.nl/trialreg/admin/rctview.asp?TC=6395 (Archived by WebCite at http://www.webcitation.org/71nc8jzwM)

**Registered Report Identifier:**

RR1-10.2196/10193

## Introduction

### Background

Over the past decades, patient involvement in decision making and delivery of health care has become increasingly important to patients, health care providers (HCPs), and policy makers. Patient involvement is pursued because of autonomy principles, as an essential element of patient-centered care and as a means to improve the quality and efficiency of care [1-4].

Personal health records (PHRs) are tools that have been developed to facilitate patient involvement in decision making, disease management, and care coordination [5]. PHRs are electronic health records that are, in varying degrees, controlled by patients [6]. Standalone PHRs are completely managed by patients [6]. Patients may use them as their personal Web-based archive for storing documents about their health; for tracking and monitoring health data; and for sharing information with HCPs, family, or caregivers. Some standalone PHRs are interoperable with a particular HCP information system, and they may be used to access medical records that are maintained by HCPs [6]. In contrast, tethered PHRs, also called patient portals, are extensions of HCP electronic medical records where most data are maintained by HCPs. Patients can use tethered PHRs to view personal health information in their records; however, usually, they cannot enter data [6]. Through a tethered PHR or interoperable standalone PHR, patients can usually view a limited set of data, often including the problem list, medication list, allergies, test results, and, less frequently, consultation notes [7,8].

Although PHRs that allow patients access to personal health information are promising tools, evidence of their effects on patient-centered care, efficiency of care, and health outcomes is inconsistent [7,9,10]. In addition, adoption rates among patients vary greatly. A recent systematic review has reported that the adoption rates of patient access to summary records range from 9% to 69% in primary care in real-life experiments in the United States [11,12]. In Europe, the adoption rates of patient access to summary records through national systems in the United Kingdom and France have been low at 0.5% and 1.5%, respectively [13,14]. However, in Sweden, 38% of the population had adopted access to the medical records in primary and specialist care in 2017 [15].

In the Netherlands, electronic health records are widely used in general practice and specialist care. A national electronic health record system is not available. However, there is a vast variation in health information systems that are used, and most of these systems do not interoperate with each other. General practitioners (GPs) have a central role in the fragmented Dutch health care system, and they receive information from all HCPs who are involved with each of their patients and store this information in patients’ general practice records (GP records). In 2016, a law was passed obliging HCPs to provide their patients digital access to their medical records by 2020. Accordingly, Web-based access to medical records is increasingly being offered to patients, mainly through patient portals. In 2017, 30% of medical specialists offered Web-based access to the list of diagnoses and test results, and almost 25% of GPs allowed their patients Web-based access to their medication list. However, only few GPs offered access to other parts of medical records: 12% to the list of diagnoses, 11% to test results, and 3% to consultation notes [16]. The adoption of these services by patients is still low. In 2017, less than 10% of patients with a chronic disease actually accessed parts of their medical records, which may be partially explained by their lack of awareness about these services [16]. Standalone PHRs are rarely used in the Netherlands; in 2016, only 1% of the population used these records [17].

MijnZorgnet is a Web-based, noncommercial, standalone PHR that provides patients a secure environment to store and share health data. In the past, the PHR has been made interoperable with the electronic health records used in fertility care to study the effects of Web-based access to medical records. Patients reported that they found the PHR useful; however, this study did not demonstrate an effect on empowerment related to use of the PHR [18,19]. In addition, the PHR has been used and evaluated in maternity care. This study demonstrated an adoption rate of 4%, which was explained by the low perceived usefulness of the PHR by healthy women with uncomplicated pregnancies. The authors suggested that the PHR might be more useful if it would be embedded in standard care [20,21]. Searching for new ways to facilitate patient involvement and foster patient-centered care, MijnZorgnet has recently been made interoperable with the infrastructure of the Dutch National Connection Point, which is used for the exchange of summary records between GPs and out-of-hour GP services and of medication lists between GPs and pharmacies. The connection between MijnZorgnet and the National Connection Point provides an opportunity to offer patients Web-based access to the summary of their medical records in general practice and to explore whether patients are interested in using this service and if and how they may benefit from this. In this paper, we have described the protocol that we will use for our study to explore the adoption and effects of patient access to the summary of their GP records through the MijnZorgnet PHR.

### Objectives

The aim of our study is to explore the use, experiences, and effects of patient access to the summary of GP records through the MijnZorgnet PHR. We intend to explore the barriers and facilitators for the adoption of PHR and Web-based access to GP records and investigate whether patients consider the information in the summary of their GP records useful. Furthermore, we aim to investigate whether access to GP records through PHR fosters patient involvement in their care and aim to explore the perceptions of GP staff regarding patients’ Web-based access to their records and patients managing their own PHRs.

## Methods

### Conceptual Framework

**Figure 1:**
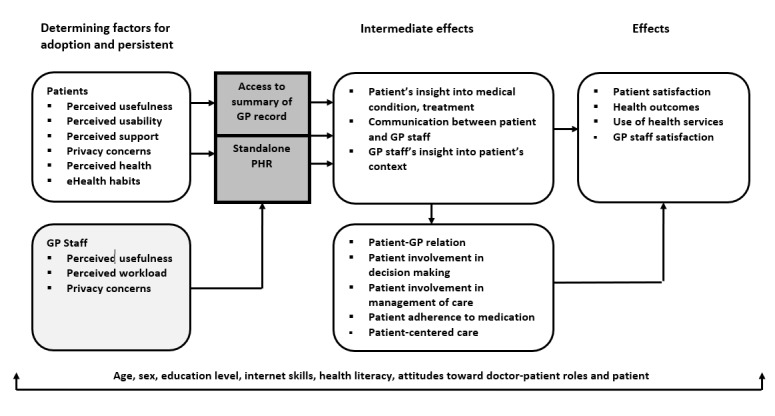
Conceptual framework. GP: general practitioner; GP staff/record: general practice staff/record; PHR: personal health record.

The conceptual framework of our study is summarized in Figure 1. We distinguished two components of the intervention: the standalone PHR and access to the summary of the GP record. We expect that access to the summary record may increase patients’ knowledge and understanding of their conditions and treatments [7,22,23]. In addition, we assume that the standalone PHR may help patients monitor their health and that the messaging function of the PHR will provide them with another means to communicate with their GPs. We expect that GPs’ and practice nurses’ insight into their patients’ symptoms, social context, and treatment plans advised by other HCPs will increase when they are invited by patients to view information stored in their PHRs.

Drawing on the Unified Theory of Acceptance and Use of Technology, we assumed that perceived performance (usefulness) of the PHR and access to GP records, perceived efforts needed to use the PHR and access GP records (usability), and social influence are the determining factors for the uptake and continuous use of the two components of the intervention [24]. Based on studies about patient portals, we added perceived health, presence of a chronic disease, eHealth habits, and concerns about privacy as the determining factors [25-27]. Moreover, we considered perceived support from the GP for using the intervention as a social influence. Following Snyder’s model on patient involvement, we assumed that an increased insight into patients’ medical situation and improved communication may enhance patient involvement and improve the doctor-patient relationship, which may subsequently affect satisfaction with care and utilization of health services and eventually health outcomes, although we expect that the latter will need more time to change than the 6-month period of our study [1]. In addition, we expect that patients who are more involved in their care may find both components of the intervention useful; thus, they are more likely to use this intervention. We assumed that sociodemographic factors, such as age, gender, education level, ethnicity and health status, health literacy and ideas about the roles of patients and GPs, and attitudes toward patient involvement are the moderators for uptake and effects [28-31].

### Study Design

**Figure 2:**
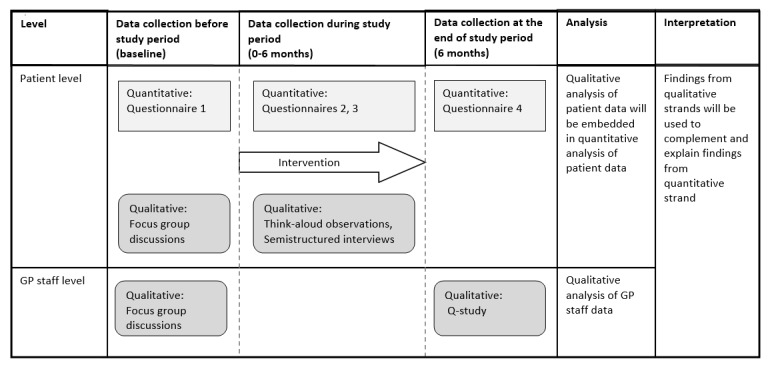
Study design. GP staff: general practice staff.

**Figure 3:**
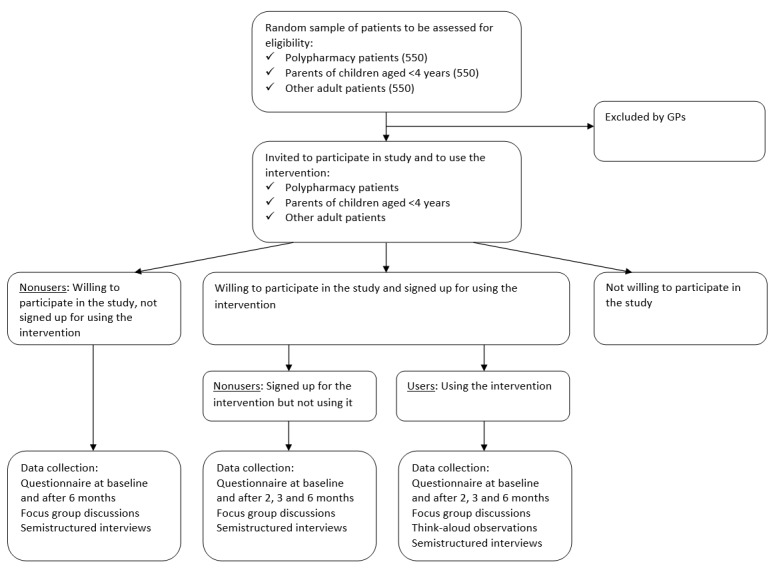
Flowchart of the study. GP: general practitioner.

We have designed a multilevel mixed-methods study in which the qualitative strand at the patient level is embedded in the quantitative before-after study at the patient level [32]. Alongside the study strands at the patient level, there will be a qualitative strand at the general practice staff (GP staff) level. All participants of the study will be offered the intervention, and data will be collected at baseline and after 6 months from patients who adopt the intervention and use it at least once during the study period to view the summary of their GP record (users) and from those who do not (nonusers). We will collect quantitative and qualitative data concurrently and use qualitative data from the earlier phases for data collection instruments that we will apply in the later phases of the study. In addition, qualitative and quantitative data will be integrated for the analysis and interpretation of the results using qualitative results to complement and explain the quantitative results. We will utilize the guidelines provided by the Good Reporting of A Mixed Methods Study [33] and Consolidated Criteria for Reporting Qualitative Research [34]. The study design is summarized in Figure 2. The flowchart of the study is presented in Figure 3.

### Intervention: MijnZorgnet Patient Health Record With Web-Based Access to the Summary of General Practice Records

MijnZorgnet is a standalone PHR where patients can store personal health information. Patients may share information with HCPs or others by inviting them to their care team at MijnZorgnet and allowing them to view their PHR. Patients can also exchange messages with the members of their care team through MijnZorgnet. To log in to the MijnZorgnet PHR, patients use their digital identity code (DigiD) with short message service verification. DigiD is the identity code that is used for government websites in the Netherlands.

The participants of this study will be able to view the summary of their GP records. This so-called “professional summary” is an automatically created set of data that was originally established to provide relevant information to HCPs working for out-of-hour GP services. The summary contains the list of medications that have been prescribed in general practice; the list of health problems, allergies, and contraindications; recent test results; and notes about the last 5 consultations with the GP, practice nurse, or practice assistant. This summary, including complete consultation notes and not including correspondence, is slightly different from the set of information that is recommended in the recently published guidelines by the Dutch College of General Practitioners and the Dutch Federation of Patient Organisations [35]. We chose to use the professional summary because this dataset is already automatically generated. In addition, drawing on the Open Notes study, we expect that access to complete consultation notes may be valuable to patients [8,36].

Patients will be able to see the summary of their record in “real time.” The information is displayed with easy-to-understand names for the different parts of the records, which have been suggested by a panel of potential users who will not participate in the study. Medical terms in the problem list and the medication list are linked to websites with evidence-based information [37,38]. Participants may copy and paste information from their GP record to their PHR and share such information with other HCPs or their family or caregivers. They will not be able to add information to their GP records; neither will they be able to access medical records from hospitals through the PHR.

The PHR and Web-based access to the summary of the GP records will be introduced to patients in a brochure that they will receive together with the invitation to participate in the study, signed by their GPs. When they log in to the PHR for the first time, they will be guided through an instruction on how to use the PHR. In addition, the participants can contact the MijnZorgnet help desk about questions regarding the PHR and how to access their summary record. The GP staff can be approached for questions about the content of the summary record.

### Setting, Study Population, and Sample Size

The study will be conducted in 3 group practices of GPs in the southeast of the Netherlands, where 18 GPs provide primary care to approximately 22,000 patients. This setting is a pragmatic choice based on the health information system that is used in these practices and the willingness of the GPs to provide Web-based access to their records and to participate in this study. Assuming that uptake, use, and effects will differ among different groups of patients, we will recruit a random sample from three groups of patients: (1) adult patients using five or more different types of medication (polypharmacy patients), (2) parents of children aged <4 years, and (3) other adult patients (those who do not belong to the first two groups). Both polypharmacy patients and parents of young children are frequent users of GP services, with an expected number of 5 and 2 contacts, respectively, with the GP during the 6-month study period [39]. They are likely to differ in terms of health status, age, internet use, and computer skills. Patients aged >75 years and those with severe cognitive or psychiatric problems will be excluded; furthermore, patients for whom access to the records may be harmful to themselves or others according to the GPs will be excluded.

Because of the lack of available figures, a proper power analysis to determine the sample size is not feasible. However, we expect that a sample of 50 users and 100 nonusers of the PHR in each group will be suitable for our aims. We assume that the uptake of the intervention will be approximately 10%, based on experiences in these practices along with the adoption rates of other eHealth interventions and based on the adoption rates of these services in the Netherlands [16]. With an expected participation rate of 25%-50% for research using questionnaires in general practice in the Netherlands [40,41] and an assumed uptake of the intervention of approximately 10% and taking into account that GPs may exclude some patients, we will use a random sample of 550 patients for each group for the quantitative study: 550 polypharmacy patients, 550 parents of children aged <4 years, and 550 other patients.

The participants of the qualitative study strand among patients will be obtained from the sample of participants of the quantitative study strand who indicate in the first questionnaire to be willing to participate in the qualitative study. To obtain a sample of patients who vary in age, gender, and education level, we will use purposive sampling. The sample size of the qualitative study strand among patients will be determined using the saturation principle. For the focus group study, we expect to achieve saturation across groups by conducting 3 focus group discussions (one for polypharmacy patients, one for parents of young children, and one for the other patients), with each group including 5-8 participants. In addition, we strive to achieve saturation within groups by inviting patients who differ in age, gender, and education level. If we feel that saturation has not been reached, we will consider conducting more focus group discussions. We will continue with think-aloud observations and semistructured interviews until we detect no new information in 3 consecutive observations or interviews.

For the study strand at GP staff level, we aim to include not only GPs but also practice nurses and practice assistants as participants to obtain a complete understanding of the use and effects of the PHR and to gain access to the summary of the GP records. Practice nurses and practice assistants also enter information in the GP records, and they may also be approached by patients with questions regarding the content of the records. Because their roles differ from those of the GP, their attitudes, experiences, and opinions concerning the PHR and access to the records may also differ, and therefore, their inclusion in the study may be useful. Sampling for the focus group study among GP staff will be performed purposively. To achieve saturation within focus groups, we will include at least two GPs, a practice nurse, a practice assistant, and a GP in training in each group. To achieve saturation across groups, we will conduct a focus group discussion with the GP staff in each of the 3 practices. All GPs, practice nurses, practice assistants, and GPs in training will participate in the Q-study at the end of the study period.

### Recruitment Procedure and Informed Consent

Eligible patients will be invited to participate in the study to create a profile on MijnZorgnet and to sign up for Web-based access to the summary of their GP records. They will receive an invitation letter signed by their GP along with the study brochure and a consent form. In the invitation letter, a code will be included that patients can use to open the first digital questionnaire. The first page of this questionnaire is a consent form. If patients indicate on this page that they do not consent to participate, they will not be able to complete the questionnaire. In the first questionnaire, patients will be asked whether they are willing to participate in the qualitative study strands. We will send a reminder after 3 and 6 weeks to all patients who have not used the code in the invitation letter.

All patients who attend a focus group session or who are interviewed or observed during the think-aloud sessions will be asked to sign a consent form at the beginning of the session. Similarly, the GP staff will be asked to sign consent forms.

To sign up for Web-based access to the summary of their GP records, patients will need to go to the GP practice to hand-in the consent form for Web-based access to their records and for identification. Subsequently, they will receive an email with a link to the PHR and a link to activate the connection between the PHR and the summary of the GP record.

### Outcomes of Interest

The primary outcomes of interest are the predictors for adoption of the PHR, which are defined as using the PHR at least once to access the summary of the GP record, including patient characteristics and perceived barriers and facilitators. Other primary outcomes are those related to patient involvement: playing an active in role decision making, disease management, and medication adherence. We included medication adherence because this is related to the knowledge about the condition and treatment and shared decision making and may be facilitated by having an overview of the medication list [42,43].

The secondary outcomes are patient-reported changes in use of GP services (number and type of contacts), patients’ confidence in their communication with the GP, knowledge about the disease, satisfaction with GP care, and patient-reported perception of changes in the doctor-patient relationship. Patient-reported benefits and drawbacks and the opinions of the GP staff about patients using the PHR and access to the summary of the GP record are also secondary outcomes. We do not expect health outcomes to improve during the 6-month study period; therefore, we will not assess these.

### Data Collection

At the patient level, we will collect quantitative and qualitative data; at the GP staff level, we will only collect qualitative data. Data collection at the patient level is summarized in Table 1.

### Collection of Data at the Patient Level

#### Questionnaires

Participants will fill out Web-based questionnaires at baseline and after 2, 3, and 6 months (Q1, 2, 3, and 4, respectively). Q1 is the baseline questionnaire, and it includes self-generated items to assess the moderating factors that we included in the conceptual framework and a validated scale to assess health literacy, the Dutch version of the Set of Brief Screening Questions (SBSQ-D). The SBSQ-D contains 3 statements that are scored on a 5-point Likert scale ranging from 0 to 4. An average score of ≤2 indicates inadequate health literacy. In the Dutch setting, construct validity and internal consistency of the SBSQ-D have been found to be adequate, and the latter has a Cronbach alpha coefficient value of 0.69 [44,45]. To assess the determining factors of perceived usefulness and benefits, security and privacy concerns, and eHealth habits, Q1 includes some self-generated questions, which are formulated as statements to which the extent of agreement may be scored on a 5-point Likert scale. An item from the Dutch Consumer Quality index for general practice is included to assess the determining factor perceived health [46]. Furthermore, Q1 contains validated scales that measure the baseline levels of the (intermediate) effects. We will use the Partners in Health scale (PIH) to assess the effects on patients’ perceived knowledge about their conditions and treatment (2 items), playing an active role in their treatment (4 items), and recognition and management of symptoms (2 items) [47]. This scale has been developed in Australia to assess patients’ self-management skills. It has been used in the Netherlands among ambulatory patients with chronic diseases. Moreover, it demonstrated adequate internal consistency, Cronbach alpha 0.69 for active role, 0.89 for knowledge, and 0.66 for the recognition and management of symptoms [47,48]. Responsiveness has been demonstrated in ambulatory patients with chronic asthma in the United Kingdom and ambulatory patients with osteoarthritis in Australia [49,50]. Items are rated on a 9-point scale and averaged for each domain, with higher scores referring to, for example, a more active role. To the best of our knowledge, the PIH has not been used among the parents of young children. We adjusted the phrasing of the items for the parents and piloted the rephrased items among them. The Medication Adherence Report Scale (MARS-5) [51] is used to assess self-reported attitudes and behavior regarding medication adherence [51]. The scale contains 4 items about intentional adherence and one item about nonintentional nonadherence. The frequencies of nonadherent behavior are rated on a 5-point Likert scale (1: very often to 5: never), and the scores are summed up for either all 5 items or for the items of intentional and nonintentional nonadherence separately. The scale has been used in various studies in the Netherlands and has shown good internal consistency (Cronbach alpha approximately 0.80) [52-54]. Construct validity has been found to be inconsistent compared with objective measures for medication adherence, which may be related to anonymous or nonanonymous use of the scale [53-55]. Because we are interested in intentions to adhere rather than actual medication usage, we decided that we could use the scale in our study. The MARS-5 has been used in the Netherlands to identify changes in adherence, comparing the sum of scores over time [56].

An item from the Dutch Quality of Care Index for general practice on satisfaction with care in general practice [46] has been included to assess patient satisfaction with care. We have also included the Perceived Efficacy in Patient-Doctor Interactions scale (PEPPI-5) to measure patients’ confidence in their communication with their GPs. The 5 items of this instrument are rated on a 5-point Likert scale (1: not confident at all to 5: completely confident). The scores are summed and averaged [57,58]. Validation in a population of ambulatory patients with osteoarthritis in the Netherlands has demonstrated a high internal consistency (Cronbach alpha 0.92), fair test retest reliability, and high construct validity [57]. The PEPPI-5 has been used in the Netherlands to assess changes in the perceived confidence of patients in their interactions with HCPs over time [59,60]. We slightly changed the wording of 1 item because we expected the original phrasing to be confusing. The PEPPI-5 has not been validated among parents of young children.

In addition, Q1 contains 2 self-generated items assessing patients’ use of GP services, asking them about the number and type of contact with the GP or practice nurse within the last 3 months. Q1 has been piloted, which has resulted in the rephrasing of the self-generated question on privacy and security concerns as well as the self-generated question on perceived usefulness.

**Table 1:** Data collection at the patient level.

Data to be collected		Quantitative data collection methods (point of time)	Qualitative data collection methods (point of time)
**Moderating factors for uptake, use, and effects**			
	Age, sex, education level, internet use, and skills	Q1^a^ (baseline)	—
	Health literacy	Q1 (baseline): SBSQ-D^b^	—
	Attitude toward patient-doctor roles and patient involvement	Q1 (baseline)	Focus group discussions (baseline); interviews (2-6 months)
**Determining factors for uptake**			
	Expected usefulness; expected usability; concerns about privacy; perceived support from the general practitioner (GP)	Q1 (baseline)	Focus group discussions (baseline)
	Perceived health; presence of a chronic disease	Q1 (baseline)	—
**Use (reach, dosage, and fidelity)**			
	Personal health record (PHR) used to store data; PHR used to share data	Q2, Q3, and Q4^a^ (2, 3, and 6 months, respectively)	Interviews (2-6 months)
	Summary of general practice records (GP records) accessed	Log data: number of hit days during the study period (6 months); Q2, Q3, and Q4 (2, 3, and 6 months, respectively)	Interviews (2-6 months)
**Experiences with PHR and access to the summary of GP records**			
	Experienced barriers and facilitators; experienced usability; experienced usefulness, benefits, and drawbacks	Q2, Q3, and Q4 (2, 3, and 6 months, respectively)	Think-aloud observations (1-3 months); interviews (2-6 months)
**Primary outcomes: changes in the following**			
	Active role in decision making	Q1, Q4: PIH^c^ (baseline, 6 months)	Interviews (2-6 months)
	Active role in care delivery	Q1, Q4: PIH (baseline, 6 months)	Interviews (2-6 months)
	Medication adherence	Q1, Q4: MARS-5^d^ (baseline, 6 months)	Interviews (2-6 months)
**Secondary outcomes: changes in the following**			
	Knowledge about the disease and treatment	Q1, Q4: PIH (baseline, 6 months)	Interviews (2-6 months)
	Confidence in communication with the GP	Q1, Q4: PEPPI-5^e^ (baseline, 6 months)	Interviews (2-6 months)
	Satisfaction with GP care	Q1, Q4 (baseline, 6 months)	Interviews (2-6 months)
	Patient-GP relationship	Q4 (6 months)	Interviews (2-6 months)
	Use of GP services	Q1, Q4 (baseline, 6 months)	Interviews (2-6 months)

^a^Q1, Q2, Q3, and Q4: questionnaires 1, 2, 3, and 4, respectively.

^b^SBSQ-D: Dutch version of the Set of Brief Screening Questions.

^c^PIH: Partner in Health scale.

^d^MARS-5: Medication Adherence Report Scale.

^e^PEPPI-5: Perceived Efficacy in Patient-Doctor Interactions scale.

Q2 and Q3 are brief, containing 6 self-generated questions to check whether participants are able to navigate through the PHR, medication list, problem list, and consultation notes and are able to comment on the completeness and usefulness of the information. These questions are formulated as statements to which participants can express their agreement on a 5-point Likert scale. In addition, 2 open questions will be added asking how patients feel about using the PHR and about accessing the summary of their GP records.

Q4 is similar to Q1; it includes the same validated scales to assess for intermediate effects, allowing us to assess differences over time. It also contains self-generated items on the usability of the system and on usefulness of the PHR and information on the summary of the GP record and use of GP services, which are formulated as statements with a 5-point Likert scale to express patients’ agreement or disagreement.

#### Log Data and Page Views

Log data on the number of days during the 6-month study period when the patients log in to the system (hit days in 6 months) and view their medication list, problem list, test results, and consultation notes will be collected to assess the actual utilization of the PHR to access the summary of GP records.

#### Routine Patient Data From General Practices

To describe our sample of study participants in relation to the population that the sample was obtained from, we will collect routine data (age, sex, prevalence of chronic disease, and frequency and number of contacts with the GP or practice nurse) from GP practices for the groups of patients from which we have obtained our samples.

#### Focus Group Discussions

Before the implementation of the intervention, we will conduct a focus group discussion with each of the 3 patient groups who participate in the study, for example, one with polypharmacy patients, one with parents of young children, and one with other adult patients. The sessions will start with a brief explanation about PHRs in general and MijnZorgnet specifically using a short movie. Subsequently, we will show the PHR and the pages that the patients will be able to view in their medical records in general practice. We will provide enough time for answering questions about the PHR and access to medical records and the National Connection Point. We will use a predefined topic list based on our conceptual framework to guide the discussion, including expectations about the usefulness of keeping a PHR and viewing the professional summary of the GP records and ideas about patient involvement in care and concerns about privacy and security. Two investigators will moderate the sessions, which will be audiotaped.

#### Think-Aloud Observation and Introspection

We will use the think-aloud method to assess the usability and usefulness of the MijnZorgnet PHR and avoid reporting bias [61-64]. We will ask patients to verbalize their thoughts while they perform predefined tasks, such as checking their medication list in their GP records. We will complement the think-aloud method with introspection [63], asking participants to explain why they performed tasks the way they did. To assess the extent to which information in the GP record is clear, understandable, desirable, and valuable to them, we will ask participants to comment on the information that they view in their GP records [65]. The think-aloud sessions will be conducted with patients who access their GP records for the first time through the PHR, and those who have logged in before. We will explain the method to them at the start of the session. Think-aloud observations and interviews will be audiotaped.

#### Semistructured Interviews

We will conduct semistructured interviews to explore patients’ experiences, opinions, and concerns regarding the PHR and access to the summary of the GP records. A topic list has been established based on our conceptual framework, including barriers and facilitators for using the PHR and access to the summary GP record, usability and usefulness of the PHR to store and share information, experienced usefulness (understandability, credibility, and desirability) of the information in the GP records, experienced benefits and drawbacks related to the use of the PHR and accessing the summary GP record, experienced changes in relation with the GP, involvement in decision making and disease management, and patient-centeredness of care. The interviews will be conducted over a period of 5 months, which allows us to obtain information from more and less experienced patients. The interviews will be audiotaped.

### Collection of Data at the General Practice Staff Level

#### Focus Group Discussions

We will conduct a focus group discussion with GPs, practice nurses, and practice assistants in each of the 3 centers for general practice before the implementation of the intervention. The topic list for these focus group discussions contains the perceptions of the GP staff about introducing a PHR and access to the summary of GP records regarding reporting habits, work load, relationship with patients, and ideas and concerns about confidentiality. The focus group discussions will be moderated by two researchers. The sessions will be audiotaped.

#### Ranking of Statements Using Q-Methodology

At the end of the study period, we will explore the opinions and concerns of the GP staff about the usefulness, benefits, and drawbacks of patient access to the professional summary of their record through the standalone PHR using Q-methodology [66-68]. Q-methodology is primarily a qualitative research method. However, it is a combination of quantitative and qualitative techniques used to identify shared opinions. Similar to other qualitative research methods, purposive sampling is used to obtain a sample of participants with variations in potentially relevant characteristics, for example, age or profession. Participants of a Q-study rank a set of statements on a response grid according to the extent they agree (+1 to +5), feel neutral (0), or disagree with each statement (−1 to −5). Using factor analysis (quantitative technique), the patterns of opinions among participants who share characteristics may be revealed [66,67]. We will formulate statements based on findings from the focus group discussions, think-aloud interviews, semistructured interviews, questionnaires, and literature.

### Analysis

#### Analysis of Quantitative Data at the Patient Level

Descriptive statistics will be used to describe the characteristics of the users and nonusers for each group of participants. To assess whether nonusing and using responders are representative of polypharmacy patients, parents of young children, and other patients in the 3 practices, we will compare their characteristics with those of the population they were obtained from using two sample *t* tests for continuous variables and chi-square tests for categorical variables. We will perform multiple linear regression analyses to investigate the effects of age, sex, education level, perceived health, health literacy, and internet and eHealth use on the adoption of the PHR and access to the summary record, defined as having logged in at least once to the PHR and the summary record. We will use descriptive analyses for data derived from the questionnaires about perceived usefulness, experiences, opinions, and concerns and for log in data on the number of days (hit days) the users have accessed their GP records and viewed different parts of the records during the study period. For each group of patients, we will compare the means changes in in-person levels of playing an active role in decision making and in the management of condition and medication adherence over the 6-month study period between users and nonusers using two sample *t* tests. Similarly, we will analyze the secondary outcomes. We will use a *P* value <0.05 (two sided) as a criterion for statistical significance for all analyses. Because of the explorative character of our study, we will not correct for multiple testing using a more stringent *P* level. We will deal with the problem regarding multiple testing in the interpretation of results by primarily focusing on the primary outcomes and by integrating the qualitative findings with the results of quantitative comparisons. SPSS Statistics software (IBM Corp, Armonk, NY, United States) will be used for the analyses.

#### Analysis of Qualitative Data at the Patient Level

Focus group discussions, think-aloud observations, and semistructured interviews will be transcribed verbatim. Using the ATLAS.ti software (ATLAS.ti Scientific Software Development GmBH, Berlin, Germany), two researchers will code the data independently using open coding as well as coding within the predetermined topics: barriers and facilitators for uptake as well as concerns about privacy and security, usability, usefulness, benefits, and disadvantages. Within the topic usability, we will explore the accessibility of the system and ease to navigate through the PHR and the summary of the GP records. We define usefulness of access to the summary records as the understandability, credibility, and desirability of the information that was viewed. We consider usefulness of the PHR as the extent to which patients value the functionalities to store and share information through the PHR. We consider benefits as positive experiences resulting from the use of the PHR and accessing the summary record. The codes will be categorized into themes that will be defined by the research team in an iterative process. Using the framework approach for the analysis of qualitative data [69,70], we will search for patterns that we will use to complement and interpret our quantitative findings on fidelity, dose, reach, and effects.

#### Analysis of the Qualitative Data at the General Practice Staff Level

The focus group discussions with the GP staff will be transcribed verbatim and coded independently by two researchers. In an iterative process, the codes will be categorized and the themes and subthemes and potential patterns will be distinguished. We will analyze the Q-sorts using the PQMethod software (PQMethod, GNU GPL, Peter Schmolck, Munich, Germany). To identify the shared views among the groups who ranked the statements similarly, a by-person factor analysis of the Q-sorts will be performed. Of each of the resulting factors, a composite Q-sort will be produced, representing the ranked statements of a hypothetical person with a 100% factor loading on this factor. Two researchers will analyze the composite Q-sorts (factor arrays), comparing the statements that are ranked in the extremes of the grids and in the more neutral positions between the different factor arrays. Together, they will interpret identified shared views between certain groups of participants. The results of the focus group analysis and Q-methodology analysis will be used to complement findings at the patient level.

### Ethical Approval

Ethical approval has been requested and granted under file number 2016-2942 by the research ethics committee of the Radboud University Nijmegen Medical Center based on the Dutch Code of Conduct for Health Research, the Dutch Code of Conduct for Responsible Use, the Dutch Personal Data Protection Act, and the Medical Treatment Agreement Act.

## Results

Enrollment was completed in May 2017 and the possibility to view GP records through the PHR was implemented in December 2017. Data analysis is currently underway and the first results are expected to be submitted for publication in autumn 2019.

## Discussion

### Relevance

In this paper, we have described the protocol for studying patient access to GP records through a standalone PHR. To obtain a comprehensive understanding of the effects of this complex intervention, we designed a mixed-methods study of patients in general practice and GP staff, allowing us to assess the facilitators and barriers for the adoption of the intervention, actual use (reach, dose, and fidelity) of the intervention, and experiences and effects on patient involvement. To the best of our knowledge, this is the first study in the Netherlands to investigate patient Web-based access to summary records and use of a PHR in general practice. In addition, few studies on this topic have been carried out in Europe [71-74]. The uptake and effects of and experiences with Web-based access to medical records have been explored more extensively in the United States [26,36,75,76]. However, because the barriers and facilitators for the uptake and effects of Web-based access to medical records are likely to be determined using different factors, including social and cultural, the findings from studies conducted in the United States may not be applicable to the European context.

### Strengths and Limitations

Our protocol has some strengths and limitations. We consider the use of a clear conceptual framework as a strength of the study, even though drawing on two theories (the Unified Theory of Acceptance and Use of Technology and Snyder’s model on patient involvement), it is a rather complex framework [1,25]. This framework has helped us include all variables that are likely to be relevant to our study, and it will also guide the interpretation of findings. The mixed-methods design enables us to assess the uptake and potential effects and obtain a deeper understanding of the use and nonuse of the intervention and the effects or absence of the effects [32]. Another strength is that we will conduct the study at two levels, patient level and GP staff level. GP staff is likely to influence the use and impact of the intervention among patients. Because the relationship between patients and GP staff may change due to the intervention, it is important to obtain information from both patients and GP staff. Another strong point of this study is the use of validated instruments to assess the effects of the intervention. However, these instruments have not been validated among parents of young children. Therefore, we will need to interpret the results based on the questionnaires on the effects on involvement-related outcomes in parents with great care. Multiple testing may be another limitation of our study. We will deal with this in the interpretation of our results by focusing on the primary outcomes and aligning qualitative findings with the results of the comparisons. We will conduct this study in the practices of GPs who are open to innovation and willing to provide their patients access to their records. We are aware that this setting is not representative of Dutch general practice. In addition, we are aware that our findings about adoption will need to be interpreted with caution because our participants, particularly those who take part in the focus group discussions, think-aloud observations, or semistructured interviews, will receive more information about the PHR and its use, than is likely to occur in a real-life setting. Furthermore, we realize that not including patients older than 75 years may also influence our findings. Obviously, we will take this into account during the interpretation of our results.

### Implications for Clinical Practice or Further Research

We expect this proof-of-principle study to be useful for policy makers, patient organizations, and HCPs who want to increase patient-centered care and patient involvement through PHRs or portals that provide patients access to medical records. Further research will be necessary to assess the uptake, use, and effects of patients’ access to different parts of their medical records using PHRs on their health outcomes in various European settings.

### Conclusion

We described the protocol of a study that will be used to explore the uptake, use, and effects of patients’ access to the summary of their GP records, through a standalone PHR, on their involvement and will be conducted among GP staff, polypharmacy patients, parents of young children, and other adult patients in the Netherlands. Taking into account the complexity of the intervention, we designed a mixed-methods study that will allow us to assess for the reach, dose, fidelity, and potential effects of using PHR on patient involvement in their care. The findings of this study will add to the existing knowledge about the implementation of PHRs and Web-based access to records in primary care and, therefore, are likely to be useful for HCPs, patient organizations, and policy makers.

## Abbreviations

DigiD: Digital identification code
GP: general practitioner
GP records: general practice records
GP staff: general practice staff
HCP: health care provider
PEPPI-5: Perceived Efficacy in Patient-Doctor Interactions scale
PHR: personal health record
PIH: Partners in Health scale
MARS-5: Medication Adherence Report Scale
SBSQ-D: Dutch version of the Set of Brief Screening Questions
